# STAT3, MYC, and EBNA1 cooperate through a ZC3H18 transcriptional network to regulate survival and proliferation of EBV-positive lymphomas

**DOI:** 10.1371/journal.ppat.1013166

**Published:** 2025-05-12

**Authors:** Huanzhou Xu, Siva Koganti, Chenglong Li, Michael T. McIntosh, Sumita Bhaduri-McIntosh

**Affiliations:** 1 Division of Infectious Diseases, Department of Pediatrics, University of Florida, Gainesville, Florida, United States of America; 2 Department of Medicinal Chemistry, College of Pharmacy, University of Florida, Gainesville, Florida, United States of America; 3 Child Health Research Institute, Department of Pediatrics, University of Florida, Gainesville, Florida, United States of America; 4 Department of Molecular Genetics and Microbiology, University of Florida, Gainesville, Florida, United States of America; University of Zurich, SWITZERLAND

## Abstract

Epstein-Barr virus (EBV), a common gamma-herpesvirus linked to various malignancies, exploits host cellular mechanisms to promote oncogenesis. Our previous research identified the zinc finger protein ZC3H18 as a novel component of the cellular DNA replication machinery in the context of EBV-driven tumorigenesis. We now demonstrate that ZC3H18 expression is upregulated in EBV-transformed and cancer cell lines, as well as in EBV-positive diffuse large B-cell lymphomas from AIDS patients, compared to their EBV-negative counterparts, supporting its activation by EBV. Our experiments show that ZC3H18 expression is regulated by the key oncogenic factors STAT3 and MYC, as well as the essential viral protein EBNA1. Using inhibitors and genetic knockdown, we find that suppressing STAT3, MYC, or EBNA1 leads to decreased ZC3H18 levels, reduced cell viability, and increased apoptosis in EBV-positive B lymphoma cells. Furthermore, ZC3H18, STAT3, MYC, and EBNA1 mutually support each other’s expression through a complex transcriptional network. Notably also, ZC3H18 transcriptionally enhances components of the NF-κB pathway, contributing to NF-κB signaling even in the absence of the EBV oncoprotein LMP1, which is crucial for cell proliferation and survival of several EBV-associated malignancies. Our findings reveal a novel regulatory axis in EBV-positive cancer cells involving STAT3, MYC, EBNA1, & ZC3H18, also linking ZC3H18 to the NF-κB pathway independently of LMP1. The involvement of EBNA1 in this network may explain, at least in part, the preferential upregulation of ZC3H18 in EBV-associated tumors and highlights predictive and therapeutic possibilities for such cancers.

## Author summary

Epstein-Barr virus (EBV), associated with various cancers, reprograms host cellular mechanisms to promote and sustain malignancy. In this study, we reveal that the zinc finger protein ZC3H18, previously identified as a novel component of the cellular replication machinery, is transcriptionally upregulated in EBV-cancers. By using clinically relevant inhibitors to target key oncoproteins STAT3 and MYC, along with the essential viral factor EBNA1, we demonstrate that ZC3H18 is part of a complex transcriptional network involving these proteins and the NF-κB pathway, a critical driver of cell survival in many EBV-related malignancies. Our findings elucidate the mechanisms underlying ZC3H18 upregulation in EBV-cancers and suggest that simultaneously targeting ZC3H18 and its network partners may be of therapeutic interest.

## Introduction

Epstein-Barr virus (EBV) is implicated in approximately 2% of global cancer-related deaths annually, underscoring its impact on public health [1,2]. A ubiquitous gamma-herpesvirus, EBV infects nearly the entire adult population [3]. While primary EBV infection is frequently asymptomatic, the virus establishes lifelong latency in the host [4], causing cancers in some. These include Burkitt lymphomas (BL), diffuse large B-cell lymphomas (DLBCL), post-transplant lymphoproliferative diseases (PTLD or LPD), and nasopharyngeal carcinomas (NPC) [5,6]. Additionally, EBV has been linked to autoimmune diseases such as multiple sclerosis [7].

During the latent phase, Epstein-Barr virus (EBV) exhibits distinct gene expression patterns, classified as latency types 0 through III. Each latency program is characterized by the expression of specific subsets of latent viral proteins, including Epstein-Barr nuclear antigens (EBNAs) and latent membrane proteins (LMPs). Some of these are oncoproteins that are essential for establishing latency in B lymphocytes as well as driving B cell proliferation and transformation [8]. EBNA1, a key viral protein, plays a vital role in maintaining the EBV genome by ensuring replication and segregation of the viral episome during cell division. In the context of cancer, EBNA1 also influences gene expression, promoting the upregulation of genes such as *c-MYC* and *E2F1* through PI3Kδ and BRD7 [9–11]. This activation fosters an environment conducive to sustained cell growth and proliferation, thereby contributing to EBV-associated malignancies [11]. EBV-driven cell survival, proliferation, and DNA replication are also dependent on cellular oncoproteins such as MYC and STAT3 [12–16]. STAT3 is a key transcription factor involved in numerous physiologic processes including immune response, inflammation, and cell proliferation. It is activated by cytokines such as IL-6 and growth factors, typically through phosphorylation by JAK family kinases or Src kinases. Once activated, STAT3 translocates to the nucleus to promote the expression of genes involved in cell survival, proliferation, and angiogenesis [17]. In EBV-infected cells, STAT3 activation suppresses DNA damage responses, promotes viral latency, and supports tumorigenesis [15,16,18]. MYC, another critical transcription factor, regulates cell cycle progression, apoptosis, and metabolism [19]. In EBV-driven cancers, such as in Burkitt lymphoma, MYC overexpression, driven by chromosomal translocations, further enhances cell proliferation and contributes to tumorigenesis. The coordinated action of STAT3, EBNA1, and MYC establishes a pro-survival environment that sustains the growth of EBV-infected cells.

ZC3H18, a CCCH-type zinc finger protein, has emerged as a critical player in RNA processing, transcriptional regulation, and DNA repair [20,21]. It has been implicated in cancer progression by modulating RNA splicing, export, and degradation [22,23]. ZC3H18 also plays a key role in DNA damage response by binding to and activating the *BRCA1* promoter, facilitating homologous recombination repair [21]. Additionally, ZC3H18 has been identified as a novel component of the replisome in EBV-transformed cells, highlighting its involvement in DNA replication during oncogenesis [24].

Given ZC3H18’s roles in RNA processing, transcriptional regulation, and DNA replication, it likely plays a significant role in EBV-related cancers. Indeed, its presence at cellular DNA replication forks in EBV-transformed cells suggests a deeper involvement in oncogenic processes driven by viral infection. Notably also, ZC3H18 was rapidly upregulated in proliferating B cells following EBV infection, compared to immune-activated B cells [24]. These observations prompted us to investigate the abundance and regulation of ZC3H18 in EBV-positive cancer cells and cell lines. Our experiments revealed that ZC3H18 is more abundant in EBV-transformed and cancer cell lines, as well as in EBV-positive lymphomas from AIDS patients, compared to EBV-negative cancer lines and lymphomas. Remarkably, ZC3H18 is part of a transcriptional network involving STAT3, MYC, and EBNA1, which co-regulate each other and lead to increased ZC3H18 levels. Furthermore, ZC3H18 contributes to the abundance of NF-κB components, and thereby NF-κB signaling independently of LMP1, a major EBV oncoprotein known to drive NF-κB signaling. These findings highlight the complex relationships between cellular and viral oncoproteins that govern EBV latency and transformation.

## Results

### EBV^+^ Burkitt lymphoma lines, transformed B cell lines, and lymphomas abundantly express the zinc finger protein ZC3H18

**Fig 1:**
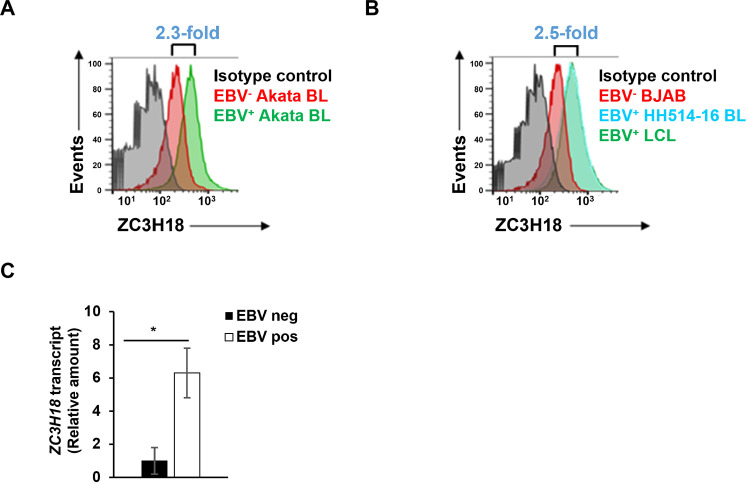
ZC3H18 is highly expressed in EBV ^**+**^ **tumor cell lines and AIDS-DLBCLs compared to their EBV**^**-**^ **counterparts.** (A, B) EBV^+^ and matched EBV^-^ Akata BL cells (A), as well as EBV^+^ LCL, EBV^+^ HH514-16 BL, and EBV^-^ BJAB B lymphoma cells (B) were seeded at 5 × 10^5^/ml. After 24 hours, one million cells were stained for ZC3H18 and analyzed by flow cytometry. Relative fold changes in ZC3H18 abundance in EBV^+^ versus EBV^-^ cells are presented. (C) RNA extracted from three EBV^+^ AIDS-DLBCL and three EBV^-^ AIDS-DLBCL tumor samples was subjected to RT-qPCR analysis using primers targeting *ZC3H18*; error bars represent SEM; *, p < 0.05.

In a previous study, we established that ZC3H18 expression is elevated in B cells newly infected with EBV compared to immune-activated B lymphocytes [24]. To address if this finding extended to EBV-positive lymphoma/transformed cells, we compared ZC3H18 levels in EBV^+^ lymphoblastoid cell lines (LCL) and EBV^+^ Burkitt lymphoma (BL) cells to those in EBV^-^ B lymphoma (BJAB) and EBV^-^ BL cells. We found a greater abundance of ZC3H18 protein in EBV^+^ tumor and transformed cell lines (Fig 1A, 1B). We further validated this observation in biopsy samples of diffuse large B cell lymphomas (DLBCL) from AIDS patients, again finding a greater abundance of *ZC3H18* transcripts in EBV^+^ compared to EBV^-^ DLBCL (Fig 1C). These findings support the idea that EBV infection induces ZC3H18 expression.

### Inhibitors of STAT3, MYC, and EBNA1 suppress intracellular levels of ZC3H18 and block cell survival and proliferation

What causes increased ZC3H18 expression in EBV^+^ cell lines and lymphomas? The cellular protooncogenes STAT3 and MYC are prominent transcription factors in EBV^+^ BL and LCL. STAT3 is rapidly phosphorylated and thereby activated following infection of primary B lymphocytes with EBV; phosphorylated STAT3 is able to translocate to the nucleus causing transcriptional upregulation of a variety of genes including *STAT3* in LCL [15]. Like LCL, latently infected BL cells demonstrate high levels of STAT3 [25,26]. As for MYC, a characteristic of BL is juxtaposition of the *c-MYC* locus on chromosome 8 into the vicinity of the regulatory elements of the immunoglobulin loci on chromosome 2, 14, or 22 [27]. Such *c-MYC*-*Ig* translocations result in overexpression of MYC. In LCL, the EBNAs drive increased expression of MYC via super-enhancer looping to the transcriptional start site of c-*MYC* [28]. While viral proteins may also regulate ZC3H18 expression, the only viral protein that is expressed in both LCL and BL is EBNA1. We therefore examined the effects of newly developed small molecule inhibitors of STAT3, MYC, and EBNA1 on ZC3H18 abundance.

**Fig 2:**
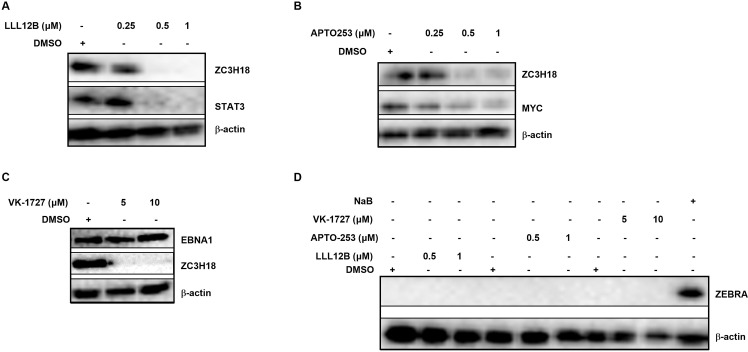
Newly developed inhibitors of STAT3, MYC, and EBNA1 suppress the abundance of ZC3H18. (A-D) HH514-16 BL cells were seeded at 2 × 10^5^/ml. After 24 hours, cells were exposed to varying concentrations of LLL12B (STAT3 inhibitor; A), APTO-253 (MYC inhibitor; B), and VK-1727 (EBNA1 inhibitor; C) for 48 hours. Cells were then collected and analyzed by immunoblotting.

The inhibitor LLL12B blocks phosphorylation, nuclear localization, and transcriptional activity of STAT3 resulting in suppressing growth and proliferation of triple negative breast cancer cells [29]. APTO-253 suppresses MYC expression by stabilizing G-quadruplex (G4) DNA structures at the c-*MYC* promoter [30]. VK-1727 interferes with EBNA1’s ability to bind DNA including the viral origin of replication (oriP) and other critical sites, thus inhibiting its functions in viral genome maintenance and cellular gene regulation [31]. As shown in Fig 2A-2C, exposure of HH514–16 BL cells to each of these inhibitors resulted in dramatic downregulation of ZC3H18 abundance. As expected, APTO-253 caused downregulation of MYC while VK-1727 did not suppress EBNA1 expression (Fig 2B, 2C). With LLL12B known to block STAT3’s transcriptional activity [29], *STAT3* gene a transcriptional target of STAT3 [15,16], and both phosphorylated and unphosphorylated STAT3 serving as transcription factors [32], we examined the effect of LLL12B on STAT3 abundance and as expected, found it to be suppressed (Fig 2A). Since STAT3 and MYC are known pro-latent factors whose suppression is critical for EBV reactivation [25,26,33], we also asked if the inhibitors triggered EBV reactivation leading to suppression of ZC3H18 expression. Conversely, might ZC3H18 suppression cause EBV reactivation? We found no evidence of ZEBRA expression, i.e. EBV lytic reactivation, when the inhibitors were used at concentrations that suppressed ZC3H18 abundance (Fig 2D). These results indicate that the observed decrease in ZC3H18 and other proteins is not attributable to lytic reactivation, implicating STAT3, MYC, and EBNA1 in regulation of ZC3H18 in EBV^+^ BL cells.

**Fig 3:**
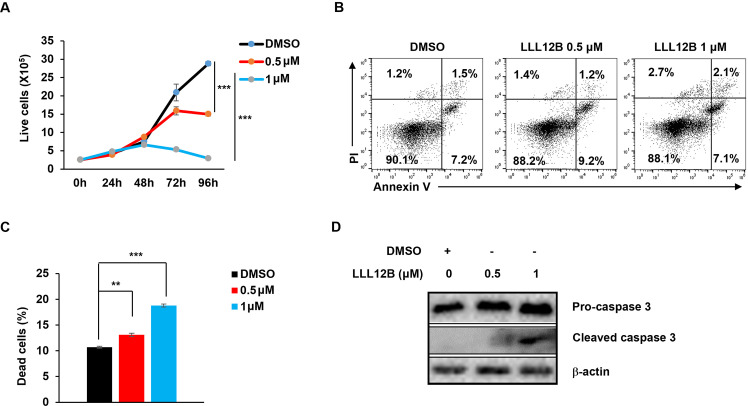
STAT3 inhibitor LLL12B impairs cell growth and induces apoptosis. (A) HH514-16 BL cells were seeded at 2 × 10^5^/ml. After 24 hours, cells were treated with different concentrations of LLL12B for the indicated times. Live cells were quantified via propidium iodide (PI) staining and flow cytometry. (B, C) Following 48-hour treatment with varying concentrations of LLL12B, HH514-16 BL cells were stained with Annexin V and PI and analyzed by flow cytometry (B). (C) Bar graph shows the percentage of dead cells; error bars in A and C represent SEM for three independent experiments; **, p < 0.01; ***, p < 0.001 (versus vehicle control); NS, not significant. (D) Immunoblot analysis of pro-caspase 3 and cleaved caspase 3 in HH514-16 BL cells exposed to LLL12B for 48 hours.

**Fig 4:**
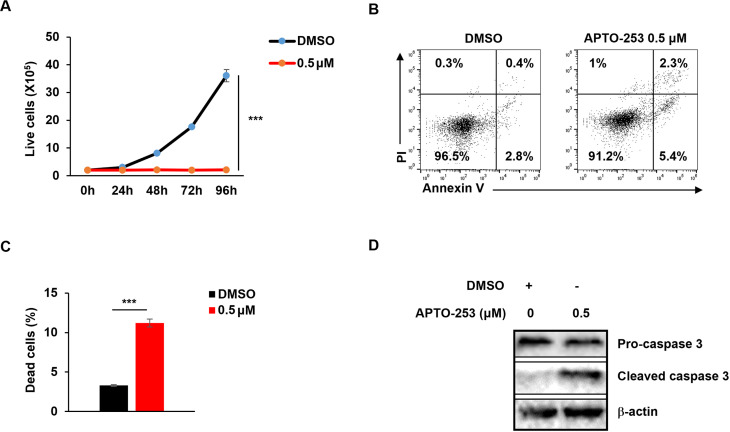
MYC inhibitor APTO-253 blocks cell growth and induces apoptosis. (A) HH514-16 BL cells were seeded at 2 × 10^5^/ml. After 24 hours, cells were treated with APTO-253 and analyzed over time by flow cytometry following PI staining to quantify live cells. (B-D) HH514-16 BL cells were treated with APTO-253 for 48 hours and stained with Annexin V and PI for FACS analysis (B, C), or immunoblotted with indicated antibodies (D). Error bars represent SEM for three independent experiments; ***, p < 0.001 (versus vehicle control).

**Fig 5:**
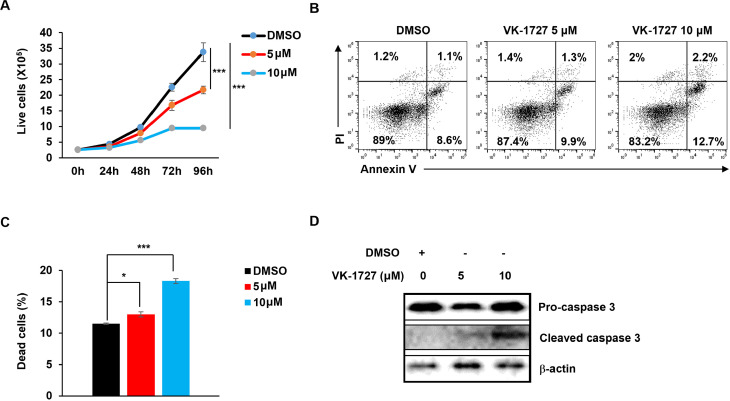
EBNA1 inhibitor VK-1727 inhibits cell growth and induces cell apoptosis. (A) HH514-16 BL cells were seeded at 2 × 10^5^/ml. After 24 hours, cells were treated with different concentrations of VK-1727 and analyzed by flow cytometry following PI staining to quantify live cells over time. (B, C) Following 48-hour treatment with varying concentrations of VK-1727, HH514-16 BL cells were stained with Annexin V and PI and analyzed by flow cytometry for apoptosis (B). (C) Bar graph shows the percentage of dead cells; error bars in A and C represent SEM for three independent experiments; *, p < 0.05; ***, p < 0.001 (versus vehicle control). (D) Immunoblot analysis of Caspase-3 and cleaved Caspase-3 in HH514-16 BL cells after 48 hours of drug treatment.

**Fig 6:**
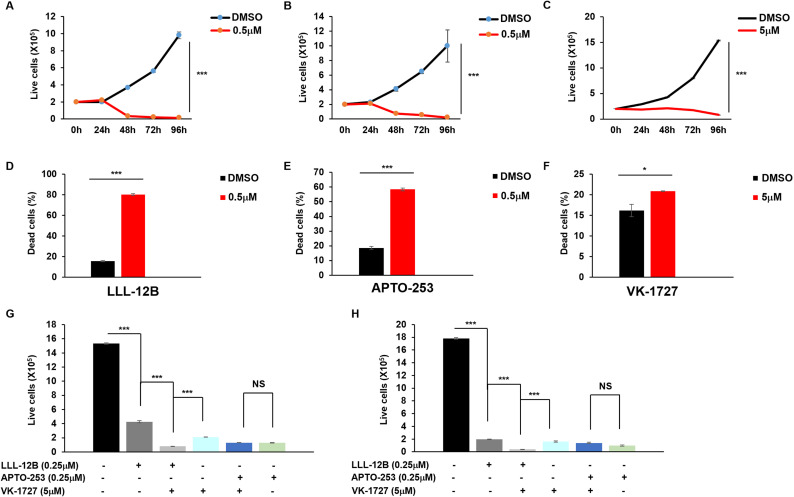
LLL12B, APTO-253, and VK-1727 inhibit cell growth and induce apoptosis in LCL. (A-F) LCL were seeded at 2 × 10^5^/ml. After 24 hours, cells treated with different concentrations of LLL12B (A, D), APTO-253 (B, E), and VK-1727 (C, F). Live cells were enumerated over time via PI staining (A-C). After 48 hours in culture, dead cells were stained with Annexin V and PI and analyzed by flow cytometry (D-F); (G-H) LCL (G) or HH514-16 BL (H) were treated with LLL12B, APTO-253, VK-1727, or combinations as indicated for 96 hours. Live cells were enumerated over time via PI staining; error bars represent SEM for three independent experiments; *, p < 0.05; ***, p < 0.001; NS, not significant.

Since STAT3, MYC, and EBNA1 are each essential for survival and proliferation of EBV-transformed and cancer cells, we tested the effects of the three inhibitors on cell viability and apoptosis in EBV^+^ HH514–16 BL cells. Our results demonstrated a significant, dose-dependent suppression of cell proliferation (Fig 3A), a concentration-dependent increase in apoptosis and cell death (Fig 3B, 3C) as well as elevated levels of cleaved caspase-3, a hallmark of apoptosis (Fig 3D) in response to LLL12B. Like the STAT3 inhibitor, both MYC and EBNA1 inhibitors caused significant reduction in the number of live cells (Figs 4A, 5A), increase in the number of apoptotic and dead cells (Figs 4B, 4C, 5B, 5C), and increased cleaved caspase-3 (Figs 4D, 5D) in BL cells. All three inhibitors also impaired survival and proliferation of LCL (Fig 6A-6F). Others have also demonstrated apoptotic death in response to EBNA-1 depletion/inhibition in nasopharyngeal cell carcinoma cells [34,35]. Furthermore, combining LLL-12B with VK-1727 reduced cell viability of LCL and BL cells even further, suggesting added benefit to targeting both STAT3 and EBNA1 (Fig 6G, 6H).

While these findings echo the effects of LLL12B on the STAT3 signaling pathway in medulloblastoma and breast cancer models [29,36,37] and APTO-253’s effects on MYC-driven proliferation of acute myelogenous leukemia, chronic lymphocytic leukemia, and myelodysplastic syndromes [38], the results from Fig 2 also suggest ZC3H18-mediated effects; we have previously shown that ZC3H18 contributes to DNA replication in EBV-transformed cells.

### STAT3, MYC, and EBNA1 regulate ZC3H18 and each other

**Fig 7:**
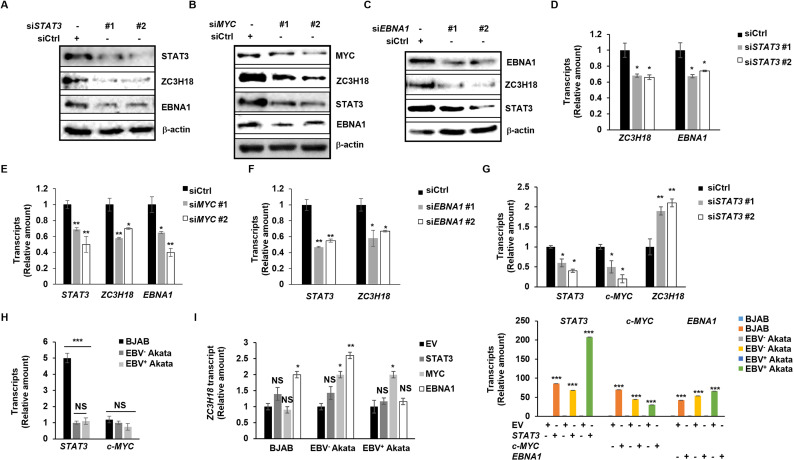
STAT3, MYC, and EBNA1 regulate ZC3H18 and each other. (A-F) One million HH514-16 BL cells were transfected with two siRNAs targeting each STAT3 (A, D), c-MYC (B, E), or *BKRF1* (EBNA1; C, F) versus control siRNA. After 24 hours, cells were collected for immunoblotting with indicated antibodies (A-C) or subjected to RT-qPCR to analyze *ZC3H18*, *STAT3*, *c-MYC*, and *EBNA1* transcript levels (D-F). (G) One million EBV^-^ BJAB cells were transfected with two siRNAs targeting STAT3 versus control siRNA. After 24 hours, cells were collected for RT-qPCR to analyze *STAT3*, *c-MYC* and *ZC3H18* transcript levels. (H) One million cells were seeded at 5 × 10^5^/ml and harvested 24 hours later. The abundance of *STAT3* and *c-MYC* transcripts was analyzed by RT-qPCR in EBV^-^ BJAB, EBV^-^ Akata BL, and EBV^+^ Akata BL cells. (I) STAT3, MYC and EBNA1 plasmids were transfected into EBV^-^ BJAB, EBV^-^ Akata BL, and EBV^+^ Akata BL cells. After 48 hours, cells were collected for RT-qPCR to assess the abundance of *ZC3H18*, *STAT3*, *c-MYC* and *EBNA1* transcripts. Error bars, SEM of 3 technical replicates; *, p < 0.05; **, p < 0.01; ***, p < 0.001 (versus control); NS, not significant; experiments were performed two to four times.

With STAT3, MYC, and EBNA1 inhibitors inducing apoptosis and downregulating ZC3H18 in both BL cells and LCL, we further explored the molecular mechanisms underlying ZC3H18 regulation. To minimize off-target effects, and importantly, toxic effects of the drugs, we used RNA interference to selectively knockdown expression of the target genes *STAT3*, *MYC*, and *EBNA1*. By depleting STAT3, MYC, and EBNA1 in HH514-16 BL cells using siRNAs, we observed substantial reductions in the levels of ZC3H18 protein and transcripts, indicating that each of these factors regulate ZC3H18 (Fig 7A-7F). Moreover, STAT3 knockdown also led to a decrease in EBNA1 transcripts and protein (Fig 7A, 7D), MYC knockdown resulted in reduced levels of STAT3 and EBNA1 levels (Fig 7B, 7E), and EBNA1 depletion caused a reduction in STAT3 transcripts and protein (Fig 7C, 7F). Since MYC is expressed from a translocated *MYC* locus in BL cells, we did not evaluate the effects on MYC in BL cells but did so in its natural context using LCL (see Fig 9 below). These results indicate potential feedback regulation between STAT3, MYC, EBNA1, and ZC3H18 in EBV^+^ cells. With ZC3H18 upregulated in EBV^+^ DLBCLs and tumor cell lines compared to EBV^-^ lymphomas and cancer lines (Fig 1), we also depleted STAT3 in BJAB cells, which are EBV^-^, and found that *MYC* transcripts were downregulated; in contrast, however, *ZC3H18* transcripts were not, supporting an EBNA1-dependent mechanism in upregulation of ZC3H18 selectively in EBV^+^ tumors (Fig 7G). The surprising upregulation of *ZC3H18* transcripts by *STAT3* siRNA suggests ZC3H18 silencing by high basal levels of STAT3 in the absence of EBNA1 in BJAB cells (Fig 7G, 7H). Moreover, suggesting a saturation effect, overexpression of STAT3 in BJAB cells failed to further regulate *ZC3H18* transcript levels (Fig 7I). To better understand the contribution of EBNA1, we overexpressed EBNA1 in BJAB (EBV^-^) cells, EBV^-^ Akata BL cells, and EBV^+^ Akata BL cells. In BJAB and EBV^-^ Akata cells, EBNA1 overexpression resulted in a significant increase in *ZC3H18* transcript levels (Fig 7I), further supporting a role for EBNA1 in the upregulation of ZC3H18. However, in EBV^+^ Akata cells, where EBNA1 is already endogenously expressed, *ZC3H18* levels remained unaltered following EBNA1 overexpression, consistent with the idea that the regulatory capacity of EBNA1 is already maximized in these cells. Of note also, STAT3 overexpression failed to further increase *ZC3H18* in both Akata cell lines while MYC overexpression resulted in upregulation of *ZC3H18* in both BL cell lines (Fig 7I). Together, depletion and overexpression experiments suggest distinct regulatory pathways for ZC3H18 in EBV^+^ and EBV^-^ contexts: in EBV^+^ cells, ZC3H18 upregulation is driven by EBNA1, STAT3, and MYC; in EBV^-^ cells, STAT3 appears to play a repressive or modulatory role. These findings highlight the intricate interplay between EBNA1, STAT3, MYC, and ZC3H18.

**Fig 8:**
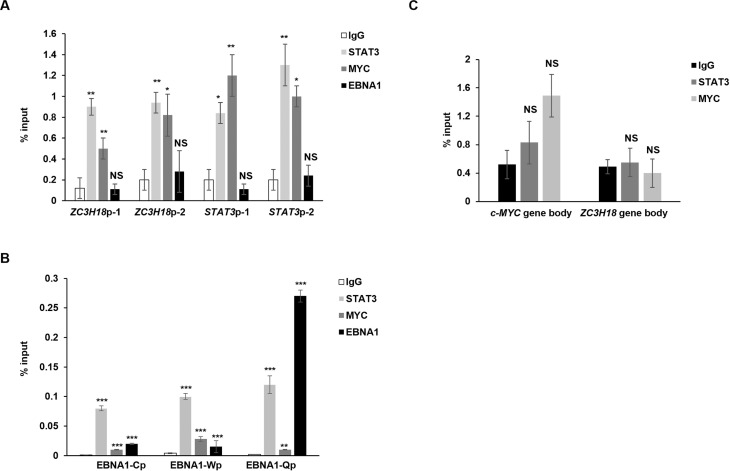
STAT3 and MYC enrich at *STAT3* and *ZC3H18* promoters while EBNA1 predominantly enriches at the *BKRF1* (EBNA1) promoter. (A-C) HH514-16 BL cells were seeded at 5 × 10^5^/ml. After 24 hours, 4 million cells were subjected to ChIP using indicated antibodies or control IgG. Precipitated DNA was subjected to qPCR analysis with primers targeting indicated promoters (A), *EBNA1* promoters Cp, Wp, or Qp (B), or gene body regions of *c-MYC* or *ZC3H18* (C). Data were normalized to input. Error bars, SEM of 3 technical replicates; *, p < 0.05; **, p < 0.01; ***, p < 0.001 (versus control); NS, not significant; experiments were performed twice.

To glean additional understanding of this coordinated regulation, we used chromatin immunoprecipitation and found STAT3 and MYC, but not EBNA1, enriched at the *ZC3H18* and *STAT3* promoters (Fig 8A). In contrast, EBNA1 enriched predominantly at the Q, and less so, C promoters of *BKRF1* (EBNA1) with enrichment also of STAT3 at these EBNA1 promoters, reinforcing the idea of a complex regulatory network between these factors (Fig 8B). To assess the specificity of these enrichments, we examined regions within the gene bodies of *c-MYC* and *ZC3H18* as negative controls. None of these negative control regions showed significant enrichment of STAT3 or c-MYC in ChIP assays (Fig 8C). Similarly, the lack of EBNA1 enrichment at *ZC3H18* and *STAT3* promoters in Fig 8A further supports the specificity of EBNA1 enrichment at the Q and C promoters observed in Fig 8B. Together, these depletion, overexpression, and ChIP experiments indicate that STAT3, MYC, and EBNA1 are not only critical for cell survival and proliferation in EBV-transformed/cancer cells, but also work in concert to regulate ZC3H18 expression and each other, positioning ZC3H18 as an effector in EBV-driven oncogenesis.

### STAT3 and EBNA1 regulate MYC

**Fig 9:**
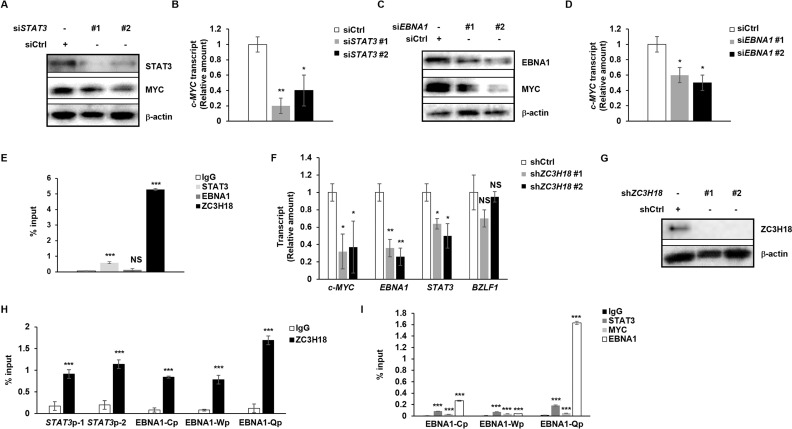
STAT3, EBNA1, and ZC3H18 regulate MYC with ZC3H18 also regulating STAT3 and EBNA1. **(A-I)** One million LCL were seeded at 5 × 10^5^/ml. After 24 hours, cells were transfected with control siRNA or two siRNAs targeting each STAT3 (A, B) or BKRF1 (EBNA1; C, D). After another 24 hours, cells were collected for immunoblotting with indicated antibodies (A, C) or RT-qPCR (B, D) to measure *c-MYC* transcript levels. (E, H) Four million LCL were subjected to ChIP with indicated antibodies or control IgG followed by qPCR with primers targeting the *c-MYC* (E), *STAT3* (H), and *EBNA1* (H) promoters. Data were normalized to IgG; error bars represent SEM of technical replicates. The experiment was performed twice. (F, G) One million LCL were transduced with lenti-shControl (shCtrl) or lenti-shZC3H18 (shZC3H18#1, #2) for 7 days and then analyzed by RT-qPCR to quantify *STAT3*, *c-MYC*, *EBNA1*, and *BZLF1* transcripts (F) or immunoblotting to confirm depletion of ZC3H18 (G). (I) Four million LCL were subjected to ChIP using indicated antibodies or control IgG. Precipitated DNA was subjected to qPCR analysis with primers targeting promoter regions of EBNA1 promoters Cp, Wp, or Qp. Data were normalized to input. Error bars, SEM of 3 technical replicates; *, p < 0.05; **, p < 0.01; ***, p < 0.001 (versus control); NS, not significant; experiments were performed twice.

To understand the effects of STAT3 and EBNA1 on MYC, we turned to LCL since MYC is expressed from the endogenous *c-MYC* locus in these cells. As in Fig 7A and 7C, we depleted LCL of STAT3 or EBNA1 using two siRNAs targeting each gene. Immunoblotting revealed reduction in MYC protein levels in each case (Fig 9A, 9C). Consistent with these findings, we also observed a significant decrease in *c-MYC* transcript levels following knockdown of STAT3 or EBNA1 (Fig 9B, 9D), further confirming that both factors contribute to maintaining MYC expression in EBV-transformed cells. Next, we performed chromatin immunoprecipitation to investigate the enrichment of STAT3 and EBNA1 at the *c-MYC* promoter. We found STAT3, but not EBNA1, enriched at the *c-MYC* promoter (Fig 9E), indicating that STAT3 likely regulates *c-MYC* transcription, whereas EBNA1 exerts its effects on *c-MYC* transcription through an indirect mechanism.

### ZC3H18 regulates STAT3, EBNA1, and MYC while also regulating NF-κB

With STAT3, MYC, and EBNA1 each regulating ZC3H18, and ZC3H18 recently discovered to be a transcription factor [21], we asked if ZC3H18 might regulate STAT3, MYC, and EBNA1. Indeed, we found that depletion of ZC3H18 resulted in significant reduction in the transcript levels of *STAT3*, *EBNA1*, and *MYC* (Fig 9F, 9G). Consistent with this observation, ZC3H18 was significantly enriched at the *MYC* promoter (Fig 9E), *STAT3* promoter (Fig 9H), and at all three EBNA1 promoters (Fig 9H). In addressing the regulation of the EBNA1/*BKRF1* promoter in LCL further, similar to BL cells, we again observed EBNA1 enrichment predominantly at Qp but less so at Cp; that said, compared to BL cells, we observed a less prominent enrichment of STAT3 at all three promoters (Fig 9I). Together, these findings suggest a feedback loop from ZC3H18 to STAT3, MYC, and EBNA1, with the last three factors also influencing each other’s expression, thereby reinforcing the transcriptional regulatory network. We note that depletion of ZC3H18 had no effect on *BZLF1* expression, i.e. disruption of latency/lytic reactivation; the EBV gene *BZLF1* encodes the lytic switch protein ZEBRA (Fig 9F).

**Fig 10:**
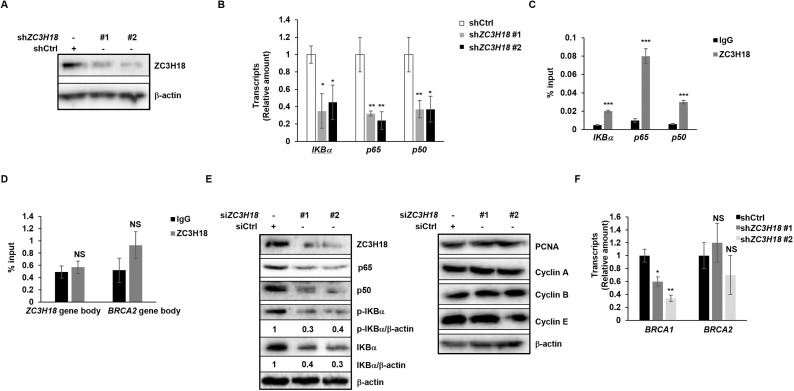
ZC3H18 regulates NF-kB components independently of LMP1. (A, B) HH514-16 BL cells were seeded at 5 × 10^5^/ml. After 24 hours, one million cells were infected with lenti-shControl (shCtrl) or lenti-sh*ZC3H18* (sh*ZC3H18*#1, #2) and selected with puromycin (5 µg/mL) for 7 days followed by immunoblotting with indicated antibodies (A) or RT-qPCR to quantify *IKBα*, *p65*, and *p50* transcript levels (B). (C, D) HH514-16 cells underwent ChIP with indicated antibodies or control IgG, followed by qPCR with primers targeting the promoter regions of *IKBα*, *p65*, and *p50* (C) or gene body regions of *ZC3H18* and *BRCA2* (D). Data were normalized to input. (E) HH514-16 cells were seeded at 5 × 10^5^/ml. After 24 hours, one million cells were transfected with two siRNAs targeting ZC3H18 or control siRNA and harvested after another 24 hours for immunoblotting with indicated antibodies. (F) HH514-16 BL cells were seeded at 5 × 10^5^/ml. After 24 hours, one million cells were infected with lenti-shControl (shCtrl) or lenti-sh*ZC3H18* (sh*ZC3H18*#1, #2) in the presence of puromycin (5 µg/mL) for 7 days and then analyzed by RT-qPCR to quantify *BRCA1* and *BRCA2* transcripts. Error bars in B, C, D, and F represent SEM of 3 technical replicates; *, p < 0.05; **, p < 0.01; ***, p < 0.001 (versus control); NS, not significant; experiments were performed twice.

Lastly, ZC3H18 was found to be important for ectopically expressed LMP1 to activate NF-κB in uninfected epithelial cells [39]. With ZC3H18 and NF-κB each known to contribute to cell survival and proliferation, we asked if ZC3H18 and NF-κB might be functionally linked even in the absence of LMP1; BL cells lack LMP1 expression. We found that depletion of ZC3H18 in BL cells resulted in significant reduction in the abundance of transcripts of key NF-κB pathway components, including *IKBα*, *p65*, and *p50* (Fig 10A, 10B). Chromatin immunoprecipitation further confirmed the enrichment of ZC3H18 at the promoters of *IKBα*, *p65*, and *p50* (Fig 10C). Further, ZC3H18 was not enriched at the gene bodies of *ZC3H18* and *BRCA2* (Fig 10D), supporting the specificity of these interactions. As expected, knockdown of ZC3H18 also resulted in reduced abundance of p65, p50, phosphorylated IKBα, and total IKBα (Fig 10E), uncovering a role for ZC3H18 in NF-κB signaling even in the absence of LMP1. To ensure that suppression of NF-κB components was not due to cell cycle disruption or cell death, we examined the levels of cyclin A, cyclin B, cyclin E, and PCNA following ZC3H18 knockdown but found them to be stable (Fig 10E). Furthermore, our earlier experiments had revealed <25% reduction in cell survival 24 hours after introduction of *ZC3H18* siRNAs [24]; using the same experimental approach here, we found a disproportionate (60–70%) reduction in the abundance of NF-kB components (Fig 10E), making it unlikely that cell death was responsible for the reduction in NF-κB components. To address if ZC3H18’s transcription related functions are independent of its replication related functions, we depleted ZC3H18 and assayed the abundance of transcripts of *BRCA1*, a gene known to be transcriptionally upregulated by ZC3H18 in ovarian cancers [21]. We found that as expected, ZC3H18 depletion resulted in a reduction in *BRCA1* transcripts but not the transcripts of the unrelated gene *BRCA2* (Fig 10F). Thus, the effects of ZC3H18 depletion on NF-κB components are not due to generalized reduction in templates resulting from ZC3H18’s functions in DNA replication but rather its role in transcription.

**Fig 11:**
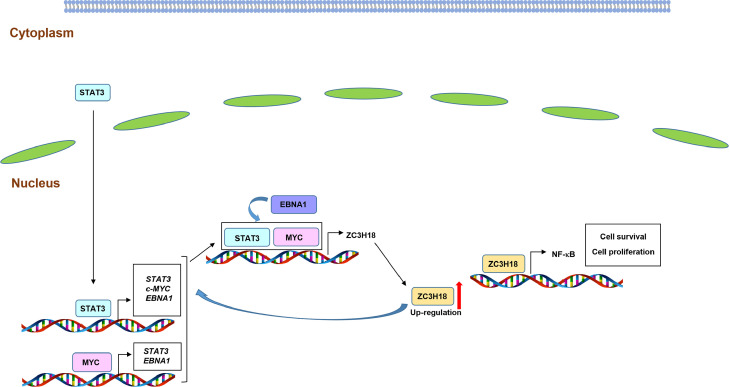
Model depicting the relationships between STAT3, MYC, EBNA1, and ZC3H18. STAT3, in EBV-infected B cells, localizes to the nucleus to regulate the expression of multiple genes, including *MYC* and *BKRF1* (EBNA1), which in turn promote the expression of the transcription factor ZC3H18. ZC3H18 transcriptionally regulates STAT3, c-MYC, and EBNA1 while also contributing to NF-κB components even in the absence of LMP1, thereby contributing to cell survival and proliferation.

In summary, our results demonstrate that STAT3, MYC, and EBNA1 collaborate to regulate the expression of ZC3H18, a protein that is upregulated in EBV^+^ lymphomas. In turn, ZC3H18 regulates STAT3, MYC, and EBNA1 while also contributing to the NF-κB pathway; a model depicting these relationships is presented in Fig 11.

## Discussion

EBV encodes potent oncoproteins, and yet, it still relies on cellular oncoproteins, some of which are transcription factors, to ensure establishment and maintenance of the latent state. This reliance on the host is especially important as, in tumor cells, the silent/latent state of EBV can take different forms depending on which and how many viral oncoproteins are expressed. Each of these forms of latency, i.e. latency types, demonstrates characteristic patterns of viral gene expression: among lymphomas, some DLBCL, in latency type III, express all the EBNAs and both LMPs, other DLBCL and Hodgkin lymphomas in latency type II, express a more restricted set of EBV proteins including EBNA1 and the LMPs, and lastly, BL, in latency type I, exhibit the most restricted pattern of gene expression limited to EBNA1. In each case, EBV depends on cellular proteins. Of those studied here, STAT3 and MYC are important in both type I and type III latency, and depletion or inhibition of these proteins causes EBV to reactivate and/or the tumor cells to die [25,26,33]. On the other hand, the NF-κB pathway is important in tumors that exhibit type III latency but is largely suppressed in BL [40] as they exhibit high MYC expression from a translocated *MYC* locus. We now show that ZC3H18 is a key cellular target of EBV that is upregulated in lymphoma lines that exhibit latency I, EBV-transformed cell lines in latency III, and EBV^+^ DLBCL in latency II/III.

ZC3H18 belongs to the family of CCCH zinc finger proteins that characteristically interact with and influence RNA metabolism [41]. However, our prior research revealed that ZC3H18 is able to interact with nascent/replicating DNA, facilitating replication of the EBV-cancer cell genome [24]. We now find that ZC3H18 is also able to enrich at promoters of prominent cellular protooncogenes and the essential EBV protein EBNA1 to transcriptionally activate those genes. Moreover, through a complex transcriptional network in which ZC3H18 itself is upregulated by each of these cellular and viral oncoproteins, ZC3H18 then contributes to a feedforward loop to support a tumor promoting environment. These observations support the premise that disrupting this network by simultaneously targeting more than one component may yield greater therapeutic promise for EBV-lymphomas (as suggested by Fig 6G, 6H). All three inhibitors studied here are the subjects of pre-clinical studies, with a derivative of VK-1727 (VK-2019) also undergoing testing in a clinical trial for nasopharyngeal cell carcinoma.

Persistent viruses successfully manipulate often disparate aspects of host biology for their own benefit. This work identifies a coordination of efforts by transcription factors, a viral replication protein, and a ribonuclear protein to benefit EBV. In particular, the involvement of EBNA1 may underlie the specific upregulation of ZC3H18 in EBV^+^ cancer/transformed cell lines and EBV^+^ DLBCL from AIDS patients. With EBV^+^ AIDS-DLBCL patients continuing to face poorer outcomes compared to EBV^-^ AIDS-DLBCL patients despite antiretroviral therapy and advanced chemotherapeutic regimens [42,43], targeting ZC3H18 alongside EBNA1 (or STAT3) may offer greater benefit. STAT3 contributes significantly to survival and proliferation of EBV^+^ cancer/transformed cells [15,16]. STAT3 and MYC are also pro-latent factors whose suppression trigger EBV lytic reactivation [25,26,33,44]. In light of this, the absence of lytic reactivation in response to the STAT3 and MYC inhibitors used here, both of which are known to block gene transcription [29,30], may be due to off target effects that curtail EBV reactivation. Of note, STAT3 also contributes to the transition from latency III to latency II, a process driven by signals from the germinal center microenvironment. Specifically, cytokines such as IL-21, secreted by T follicular helper cells, function via STAT3 to epigenetically remodel key EBV promoters [45].

ZC3H18 has previously been shown to contribute to LMP1-mediated NF-κB activation in uninfected epithelial cells [39]. Our experiments show that in an EBV-infected setting lacking LMP1 expression, ZC3H18 transcriptionally contributes to NF-κB components, not activation of NF-κB signaling. Depletion of ZC3H18 did not demonstrate a near total loss of IKBα phosphorylation but instead, showed a 30–40% reduction in both total and phosphorylated IKBα with both shRNAs (Fig 10B), supporting previous observations that to avoid apoptosis in these high MYC expressing cells, NF-κB signaling is suppressed in BL cells [40]. Others have also noted that the NF-κB p50 subunit may promote MYC stability by suppressing E2F1 transactivation of the *FBW7* gene, thereby limiting FBW7 E3 ligase-mediated proteasomal degradation of MYC [46]. In this way, ZC3H18 may contribute both to NF-κB components (thereby stabilizing MYC) and to NF-κB signaling in LMP1-expressing tumors while contributing to MYC stability in EBV^+^ BL, i.e. in the absence of LMP1. We note that in LCL, i.e. cells expressing LMP1, ZC3H18 also transcriptionally contributes to MYC.

Our overall observations, depicted in Fig 11, point to predominantly a multi-component transcriptional network in EBV-infected/cancer cells. While STAT3, MYC, and ZC3H18 transactivate each other, EBNA1 transcriptionally regulates STAT3, MYC, and ZC3H18 presumably via indirect means. However, we found EBNA1 strongly enriched at its Q promoter and to a lesser extent, the C promoter. Others have noted that EBNA1 can initiate transcription at the C promoter via a large molecular complex involving cellular transcription factors E2F1, ARID3A, and Oct-2 partially through OriP looping [11,47]. We also noted similar levels of STAT3 enrichment at all three EBNA1 promoters; however, which promoter primarily contributes to STAT3-driven EBNA1 activation is unclear. Like STAT3, we found abundant ZC3H18 enriched at all three promoters of EBNA1 in LCL; again, which promoter is predominantly activated by ZC3H18 remains to be seen. Furthermore, the relative contribution of EBNA1, STAT3, and ZC3H18 to EBNA1 expression in any single cell remains unknown and difficult to discern given the oligo/polyclonal origins of LCL. In contrast to EBV-infected/cancer cells, EBV-uninfected/cancer cells demonstrate a lower abundance ZC3H18, most likely due to the absence of EBNA1 and a STAT3-mediated repressive effect on ZC3H18.

In conclusion, our study highlights a critical role for ZC3H18 in EBV-mediated oncogenesis and its regulation by STAT3, MYC, and EBNA1. By also linking ZC3H18 to transcription of NF-κB components as well as to transcription of major cellular oncoproteins STAT3 and MYC and the essential viral protein EBNA1, we have uncovered a novel mechanism by which EBV drives cell survival and proliferation. Disrupting this network by targeting it at multiple points may offer particular benefit in the setting of EBV-associated malignancies given that EBNA1 appears to be an important contributor to this network; in contrast, lacking EBNA1, EBV-unrelated malignancies do not appear to upregulate ZC3H18 as prominently. Future studies should focus on exploring the potential for ZC3H18 as a biomarker or therapeutic target in EBV-driven cancers.

## Materials and methods

### DLBCL tumors

RNA isolated from frozen diffuse large B cell lymphomas (DLBCL), three EBV-positive and three EBV-negative, were subjected to RT-qPCR as described below. These specimens were provided by the AIDS and Cancer Specimen Resource (ACSR), funded by the National Cancer Institute. To determine if tumors were EBV-positive or EBV-negative, multiplexed PCRs were used to amplify EBNA1, LMP1, and LMP2 genes using an established assay at the Mayo Clinic based on the work of Ryan et al. [48].

### Cell lines and chemical treatment

EBV-positive BL cell line (HH514-16, a kind gift from Dr. George Miller, Yale University), EBV-positive lymphoblastoid cell lines (LCLs), and EBV-negative B lymphoma cell line (BJAB) were maintained in RPMI 1640 medium supplemented with 10% fetal bovine serum and 1% penicillin/streptomycin. All cell lines were cultured in an incubator at 37°C with 5% CO₂. For chemical treatment, cells were expanded with fresh medium and, 24 hours later, were incubated with the indicated concentrations of APTO-253 (HY-16291, MedChemExpress), VK1727 (HY-125471, MedChemExpress), or LLL12B (synthesized by Dr. Chenglong Li at the University of Florida) for 48 hours. After this, the medium was replaced, and the cells were incubated with the same chemicals for another 48 hours.

### Flow cytometric analysis of intracellular proteins

Flow cytometry was used to quantify the intracellular abundance of ZC3H18 as described previously [24]. Briefly, cells were fixed using 50 μl of Cytofix/Cytoperm solution (554722, BD Biosciences) for 15 minutes on ice. After fixation, the cells were washed twice with 1X Perm/Wash buffer (554723, BD Biosciences) and then incubated with the primary antibody (diluted in 1X Perm/Wash buffer) for 30 minutes. Cells were washed twice and then incubated with a fluorochrome-conjugated secondary antibody (diluted in 1X Perm/Wash buffer) for 30 minutes. Following two more washes, data was acquired on an Attune Nxt flow cytometer (Thermo Fisher Scientific) and analyzed using FlowJo software (FlowJo LLC). Cells stained with an isotype-matched antibody were used as control for gating.

### siRNAs, plasmids, and transfection

Transfection was performed as described previously [49]. Briefly, one million cells were seeded in a 24-well plate, and 24 hours later, transfected with 200 pmol siRNA in transfection solution (MIR50117, Mirus) using an Amaxa Nucleofector II. The siRNAs used in this study included those targeting *STAT3* (#1, 116558; #2, 116559, Thermo Fisher Scientific), *c-MYC* (#1, 103828; #2, 106820, Thermo Fisher Scientific), and *ZC3H18* (#1, 141319; #2, 141320, Thermo Fisher Scientific). The siRNA sequences targeting EBNA1 were published previously [50]; these were synthesized by Thermo Fisher Scientific.

For plasmid transfection, one million cells were seeded in a 24-well plate, and 24 hours later, 10µg of each plasmid was transfected using an Amaxa Nucleofector II. Cells were collected 48 hours after transfection. Plasmids used here included: STAT3-pEGFP-N1 (a kind gift from Dr. Nancy C. Reich at Stony Brook University, New York), FLAG-MYC (102626, Addgene [51]) and FLAG-EBNA1.

### Lentivirus production and lentiviral transduction

For lentivirus production, shControl or shZC3H18 lentiviral plasmids were co-transfected with psPAX2 (a gift from Didier Trono, 12260, Addgene), REV (12253, Addgene) [52], and pMD2.G (a gift from Didier Trono, 12259, Addgene) into 293T cells. The sequences for shRNAs targeting ZC3H18 or the non-targeting control were inserted into the pLKO.1 - TRC cloning vector (10878, Addgene) [53]. The shRNA sequences are listed in Table 1. Forty-eight hours after plasmid transfection, media were harvested, filtered through 0.45 µm syringe filters, and then lentivirus particles were concentrated using Lentivirus Concentrator (TR30025, OriGene Technologies). The lentiviruses were resuspended in RPMI-1640 medium. HH514-16 or LCL cells were transduced with lentiviruses in the presence of 8 µg/mL polybrene using spinning infection (2000 x g for 20 minutes at room temperature). Forty-eight hours after lentivirus transduction, 5 µg/mL puromycin was added to select for successfully transduced cells.

**Table 1:** Primers and shRNAs.

Primers for RT-qPCR	Forward primers (5’ ~ 3’)	Reverse primers (5’ ~ 3’)
*18S rRNA*	GTAACCCGTTGAACCCCATT	CCATCCAATCGGTAGTAGCG
*STAT3*	GAGGACTGAGCATCGAGCAGC	TTAGCCCATGTGATCTGACACCC
*EBNA1*	CTCTTAGAGAGTGGCTGCTACG	CTGGCTAAGCCTGTGACTTAGTC
*c-MYC*	CCCGGACGACGAGACCTTC	CCTGGTAGGAGGCCAGCTT
*ZC3H18*	AAGCGACCTTAGGGATGAGGC	CGCTTTCTCAGCCTCGTCC
*BRCA1*	GAAGAGCTTCCCTGCTTCCA	ACACTCGGTAGCAACGGTGC
*BRCA2*	GAATGGCAGACTGACAGTTGGT	GACTTCAAGAGGTGTACAGGC
**Primers for ChIP-qPCR**	**Forward primers (5’ ~ 3’)**	**Reverse primers (5’ ~ 3’)**
*STAT3p-1*	ACGCTTCTTCTGGAAGACCA	CG GAATG TCCTG CTGAAAAC
*STAT3p-2*	GGTATCTGGGGAACCATGTG	CTTTCC CTACCC CCATCATT
*c-MYCp-1*	CCTCTCTCGCTAATCTCCGCC	CCTTCTCGAGGCAGGAGGG
*c-MYCp-2*	TTCCAGCGAGAGGCAGAGG	GAAGCCCCCTATTCGCTCC
*ZC3H18p-1*	GCTAAAGGGCAGCCAAGGGA	TCCTGAAGCAGGAACCTGGG
*ZC3H18p-2*	ATAGCTGGGACTACAGGCGC	GCAGGTGGATCATGAGGTCAGG
*EBNA1-Wp*	CCTTCATCACCGTCGCTGAC	GTGGAGTGTTGGGCTTAGCAG
*EBNA1-Qp*	ACGAAAGTGCTTGAAAAGGCG	CCAGCTGCCCAAAATGCCA
*EBNA1-Cp*	GGACTGAAGAAACAGCCTCCTG	CAGTGCCCAGATTCATGTAAAGGG
*IKBa*	GGCTGGGGATTTCTCTGGG	CCTAGCAGAGGACGAAGCC
*P65 (RelA)*	GGTGGCCCTTGACTCAGCAT	ACTGAATCAGATGCGTTCTCCC
*P50*	CGATCCTGAAGCTCCCCTT	CGATCCTGAAGCTCCCCTT
**shRNAs**	**Sense strand (5’-3’)**	**Antisense strand (5’-3’)**
sh*ZC3H18* #1 (targets 3’UTR)	AGCACGGTTCTCATGTAAATT	AATTTACATGAGAACCGTGCT
sh*ZC3H18* #2 (targets CDS)	GGAATGAATTGTAGGTTTATA	TATAAACCTACAATTCATTCC
shCtrl	CCTAAGGTTAAGTCGCCCTCG	CGAGGGCGACTTAACCTTAGG

### Immunoblotting and antibodies

Immunoblotting was performed as described previously [54]. Briefly, cells were collected and lysed with RIPA buffer, and cell extracts were electrophoresed using SDS-PAGE and transferred onto nitrocellulose membranes. The following antibodies were used for immunostaining: rabbit anti-STAT3 antibody (4904S, Cell Signaling Technology), mouse anti-EBNA1 antibody (sc-81581, Santa Cruz Biotechnology), rabbit anti-MYC antibody (A190-105A, Bethyl Laboratories), mouse anti-β-actin antibody (clone AC-15) (A1978, Sigma-Aldrich), rabbit anti-phospho-IκBα (Ser32) antibody (2859S, Cell Signaling Technology), rabbit anti-IκBα antibody (9242S, Cell Signaling Technology), rabbit anti-p65 antibody (8242S, Cell Signaling Technology), rabbit anti-p50 antibody (13586, Cell Signaling Technology), rabbit anti-ZC3H18 antibody (A304-682A, Bethyl Laboratories), rabbit anti-Caspase 3 antibody (GTX110543, GeneTex), mouse anti-Cyclin A antibody (sc-53228, Santa Cruz Biotechnology), rabbit anti-Cyclin B antibody (4138S, Cell Signaling Technology), rabbit anti-Cyclin E antibody (A301-566, Bethyl Laboratories), mouse anti-ZEBRA antibody (sc-53904, Santa Cruz Biotechnology), rabbit anti-PCNA antibody (A300-276, Bethyl Laboratories), HRP-conjugated goat anti-mouse IgG (626520, Thermo Fisher Scientific), and HRP-conjugated goat anti-rabbit IgG (31460, Thermo Fisher Scientific).

### Assay to measure live/dead cells

Propidium iodide staining was performed as described previously to enumerate live and dead cells in unfixed samples [55]. Briefly, 2 × 10^5^ cells were seeded in a 24-well plate with medium containing the indicated concentrations of inhibitors or dimethyl sulfoxide (DMSO). Cells were spun down and resuspended in fresh medium containing inhibitors or DMSO every 48 hours. Cells were collected every 24 hours and stained with 1 μg/mL propidium iodide (PI) solution (P4864, Sigma-Aldrich) without fixation or DNA denaturation. Live cells (PI-negative) were distinguished from dead cells (PI-positive) using flow cytometry.

### Apoptosis assay by flow cytometry

Cell apoptosis was detected using the FITC Annexin V kit (556420, BD Biosciences) following manufacturer’s instructions. Briefly, one million cells were washed twice with PBS and resuspended in 1 × Binding Buffer containing 5 µL of FITC Annexin V and 1 μg/mL PI. Cells were incubated for 15 minutes at room temperature in the dark. Then, 400 µL of 1 × Binding Buffer was added to each tube, and cell apoptosis was analyzed by flow cytometry [56].

### Reverse transcriptase-quantitative PCR

RT-qPCR was performed as described previously [57]. Briefly, MuLV Reverse Transcriptase (M0253L, New England Biolabs) was used for cDNA synthesis using 1 µg RNA as template. Quantitative PCR was then used to analyze the levels of each gene. The primers used for RT-qPCR analysis are shown in Table 1. Data were analyzed using the ΔΔCT method after normalization to 18S rRNA.

### Chromatin immunoprecipitation-quantitative PCR (ChIP-qPCR)

ChIP was performed as described previously [58]. Four million cells were seeded in flasks 24 hours before ChIP. Cells were fixed with 1% formaldehyde for 15 minutes and quenched with glycine for 5 minutes. After two washes with PBS, cells were lysed in Buffer A (7006S, Cell Signaling) for 10 minutes on ice and washed with Buffer B (7007S, Cell Signaling) for 2 minutes. Lysates were resuspended in 150 µL Buffer B with 0.5 µL Micrococcal Nuclease (10011, Cell Signaling) and incubated at 37°C for 20 minutes. After centrifugation to remove the supernatant, lysates were incubated with the indicated antibodies or isotype IgG and protein G beads (9006, Cell Signaling) at 4°C overnight. Following three washes with 1X Low Salt ChIP Buffer and one wash with High Salt ChIP Buffer, DNA was purified using spin columns (14209S, Cell Signaling) according to the manufacturer’s protocol. The primers used for ChIP-qPCR are listed in Table 1.

### Statistical analysis

Two-tailed Student’s t-test was used to compare the differences between two groups of interest. The degree of significance was indicated as *, p < 0.05; **, p < 0.01; ***, p < 0.001.
