# Correction: STAT3, MYC, and EBNA1 cooperate through a ZC3H18 transcriptional network to regulate survival and proliferation of EBV-positive lymphomas

**DOI:** 10.1371/journal.ppat.1013754

**Published:** 2025-12-05

**Authors:** Huanzhou Xu, Siva Koganti, Chenglong Li, Michael T. McIntosh, Sumita Bhaduri-McIntosh

S1 Data is omitted from the list of Supporting Information. It can be viewed below.

## Supporting information

S1 Data Primary data for the results displayed as graphs. (XLSX)
