# Correction: Correction: STAT3, MYC, and EBNA1 cooperate through a ZC3H18 transcriptional network to regulate survival and proliferation of EBV-positive lymphomas

**DOI:** 10.1371/journal.ppat.1013931

**Published:** 2026-02-09

**Authors:** The PLOS Pathogens Staff

## Notice of Republication

An incorrect version of S1 Data was published in error in the correction published on December 5, 2025. This correction notice has been republished on January 20, 2026, to rectify the error. The publisher apologizes for the error.

## References

[1] Xu H, Koganti S, Li C, McIntosh MT, Bhaduri-McIntosh S. STAT3, MYC, and EBNA1 cooperate through a ZC3H18 transcriptional network to regulate survival and proliferation of EBV-positive lymphomas. PLoS Pathog. 2025;21(5):e1013166. doi: 10.1371/journal.ppat.1013166 40354417 PMC12091888

[2] Xu H, Koganti S, Li C, McIntosh MT, Bhaduri-McIntosh S. Correction: STAT3, MYC, and EBNA1 cooperate through a ZC3H18 transcriptional network to regulate survival and proliferation of EBV-positive lymphomas. PLoS Pathog. 2025;21(12):e1013754. doi: 10.1371/journal.ppat.1013754 41348692 PMC12680213

